# DNA methylation and histone post-translational modifications in atherosclerosis and a novel perspective for epigenetic therapy

**DOI:** 10.1186/s12964-023-01298-8

**Published:** 2023-11-29

**Authors:** Liang Zhang, Chenhai Xia, Yongjun Yang, Fangfang Sun, Yu Zhang, Huan Wang, Rui Liu, Ming Yuan

**Affiliations:** 1 Department of Cardiology, Xijing Hospital, Air Force Military Medical University, No. 127 Changle West Road, Xi’an, 710032 China; 2 Department of Rehabilitation, Tangdu Hospital, Air Force Military Medical University, No. 1 Xinsi Road, Xi’an 710000, China

**Keywords:** DNA methylation, Histone post-translational modifications, Epigenetic, Atherosclerosis

## Abstract

Atherosclerosis, which is a vascular pathology characterized by inflammation and plaque build-up within arterial vessel walls, acts as the important cause of most cardiovascular diseases. Except for a lipid-depository and chronic inflammatory, increasing evidences propose that epigenetic modifications are increasingly associated with atherosclerosis and are of interest from both therapeutic and biomarker perspectives. The chronic progressive nature of atherosclerosis has highlighted atherosclerosis heterogeneity and the fact that specific cell types in the complex milieu of the plaque are, by far, not the only initiators and drivers of atherosclerosis. Instead, the ubiquitous effects of cell type are tightly controlled and directed by the epigenetic signature, which, in turn, is affected by many proatherogenic stimuli, including low-density lipoprotein, proinflammatory, and physical forces of blood circulation. In this review, we summarize the role of DNA methylation and histone post-translational modifications in atherosclerosis. The future research directions and potential therapy for the management of atherosclerosis are also discussed.

Video Abstract

**Supplementary Information:**

The online version contains supplementary material available at 10.1186/s12964-023-01298-8.

## Background

Atherosclerosis is the important cause of most cardiovascular diseases, including stroke and coronary artery disease, which are the leading reasons for mortality worldwide [1]. Atherosclerosis is caused by vascular endothelial dysfunction, lipid deposition, huge phagocytosis, foam cell formation, abnormal migration, and the proliferation of vascular smooth muscle cells (VSMCs) and stromal cells under the action of promoting inflammatory factors [2, 3]. In response to pathological stimuli, such as low-density lipoprotein (LDL) and triglyceride levels, smoking, and obesity, vascular endothelial cells (ECs)—the innermost layer of blood vessels—become activated. Monocytes, a pro-inflammatory palette that can bind to adhesion molecules expressed by activated endothelial cells, are recruited into the intima through chemokine. Upon entering the intima, monocytes mature into macrophages expressing scavenger receptors and then bind lipoprotein and become differentiated into foam cells. In the progression of atherosclerotic lesions, VSMCs initially stabilize the plaque by producing extracellular matrix proteins, such as collagen and proteoglycans (reviewed in [4]). Over decades, the accumulation of foam cells, coupled with debris from dead and dying cells, results in the progression of atherosclerotic plaque. However, during the late stages of atherosclerosis, activated macrophages produce enzymes of the matrix metalloproteinases family and lead to plaque rupture, resulting in myocardial and cerebral infarctions, which significantly contribute to morbidity and mortality from atherosclerotic diseases (reviewed in [4]). Recent evidence reveals that VSMCs can also differentiate into macrophage- and mesenchymal stem cell-like cells that promote lesion expansion and instability.

Epigenetics, which was proposed by Conrad Waddington in 1942, is a phenotype involving heritability, passed on through either mitosis or meiosis [5]. Since then, epigenetics has been redefined multiple times [6]. Recently, an epigenetic trait was defined as “a stably heritable phenotype resulting from changes in a chromosome without alterations in the DNA sequence” [7]. Epigenetics has both positive and negative effects. On the one hand, epigenetic modulating agents may coordinately promote tumor immunogenicity by inducing de novo expression of transcriptionally repressed tumor-associated antigens, increasing expression of neoantigens and major histocompatibility complex (MHC) processing/presentation machinery, and activating tumor immunogenic cell death [8]. On the other hand, if exposed to environmental factors such as chemicals, drugs, stress, or infections, epigenetics is associated with the accumulation of senescent cells resulting from immune senescence [9].

**Fig. 1:**
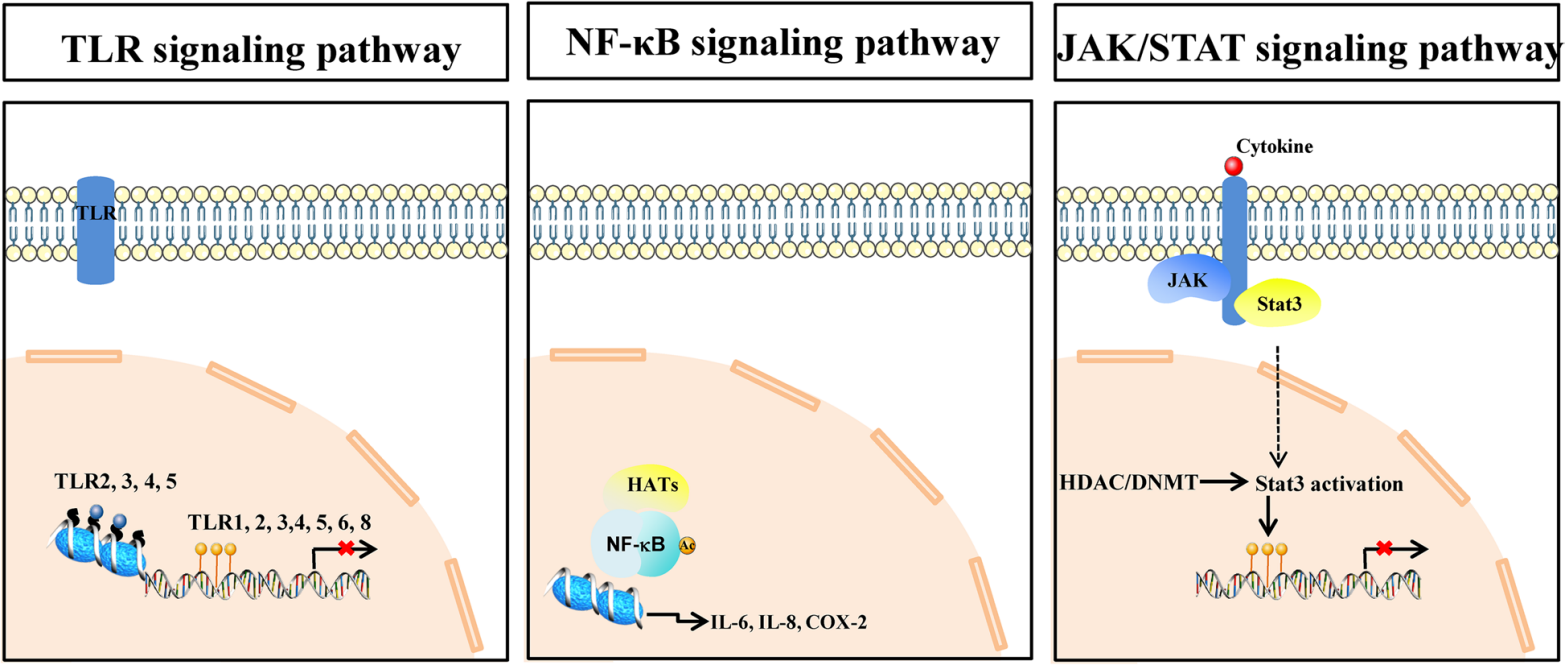
Association with epigenetic modifications and the TLR, NF-κB, and JAK/STAT signaling pathway. TLRs can be regulated by DNA methylation, and histone modifications, which will eventually result in changed TLRs expression. DNA methylation that occurs on the promoter region of TLR genes, such as TLR1, 2, 3, 4, 5, 6, and 8, can reduce the expression of TLR on the membranes. Depending on the type of modification, the histone modifications that occurs on the nucleosome near the promoter of TLR genes, such as TLR2, 3, 4, and 5, can also positively or negatively regulate the expression of TLRs on the membranes; HATs interacts directly with NF-κB or induces its acetylation, and recruits NF-κB to the promoters of proinflammatory genes such as IL-6, IL-8 and cyclooxygenase-2 (COX-2) to regulate inflammatory signaling pathway activation; JAK kinases are activated in response to stimulation, which causes Stat3 activation, resulting in Stat3 nuclear transfer. Stat3 can also be activated by HDAC or DNMT. Activation of Stat3 leading to the DNA methylation of Stat3

Recently, except for a lipid-depository and chronic inflammatory diseases, atherosclerosis is defined as an epigenetic disease [10–13]. Increasing evidences propose that epigenetic modifications are involved in the occurrence and development of atherosclerosis [14]. Microarray-based DNA methylation analysis reveales that patients with atherosclerosis have higher levels of DNA methylation than healthy controls [15]. Studies suggest that epigenetics not only regulates the expression of inflammatory cytokines but also controls epigenetic modifications through a reciprocal mechanism [16]. However, the chronic progressive nature of atherosclerosis has highlighted atherosclerosis heterogeneity and the fact that specific cell types in the complex milieu of the plaque are, by far, not the only initiators and drivers of atherosclerosis. Instead, the ubiquitous effects of cell type are tightly controlled and directed by the epigenetic signature, which, in turn, is affected by many proatherogenic stimuli, including LDL, proinflammatory cytokines, and physical forces of blood circulation. Besides, inflammatory molecular pathways (such as Toll-like receptors (TLR), NF-κB, and the JAK/STAT signaling pathway) are associated to epigenetic modifications (Fig. 1). Therefore, a better understanding of the role of epigenetics in the pathogenesis of atherosclerosis has enormous potential translational value.

In this Review, we discuss the latest major advances in our understanding of the functions of DNA methylation and histone post-translational modifications, as well as their establishment, maintenance, and erasure during atherosclerotic development. We also discuss new insights gained into the patterning of DNA methylation and histone post-translational modifications during atherosclerotic progression.

### DNA methylation in atherosclerosis

DNA methylation is one of the earliest discovered epigenetic modifications of genes. The activation or repression associated with DNA methylation goes hand in hand with the context and site of methylation [17]. DNA methylation is a dynamic and reversible modification process, which refers to the process of converting cytosine in the cytosine guanine (CG) into 5-methylcytosine (5mC) under the action of DNA methyltransferases (DNMTs) [12]. In this process, S-adenosylmethionine provides a methyl group, while DNA demethylase ten-eleven translocation protein (TET) converts 5mC into 5-hydroxymethylcytosine (5-hmC). The following are three phases of DNA methylation: establishment (de novo DNA methylation), maintenance, and demethylation. In mammals, de novo DNA methylation predominantly occurs at symmetric 5’-Cphosphate-G-3’ (CpG) sites under the action of DNMT3A/3B in an early stage of embryonal development [18]. Two major de novo DNA methylation enzymes, DNMT3A and DNMT3B [19, 20], contain a highly conserved DNMT domain (the MTase domain) in the carboxy terminus and two chromatin reading domains, ATRX-DNMT3-DNMT3L (ADD) and PWWP. Methylation maintenance enzyme DNMT1 in collaboration with another multidomain protein, E3 ubiquitin-protein ligase UHRF1, which specifically binds hemimethylated CpG dinucleotides at replication forks through its SET- and RING-associated (SRA) domain [21]. Additionally, TET methylcytosine dioxygenases (including TET1, TET2, and TET3) progressively oxidize 5mC to 5-hydroxymethylcytosine (5hmC), 5-formylcytosine (5fC) and 5-carboxylcytosine (5caC), result in activing DNA demethylation [22, 23].

Healthy individuals’ CpG islands in the promoter region of genes are normally hypomethylated, whereas the CpG islands in the non-promoter region are hypermethylated [24, 25]. Global DNA hypomethylation occurs in non-promoter regions, and it can cause the initiation of transcription at incorrect regions and high transcriptional activity in sites that are usually silent. Global DNA hypermethylation, on the other hand, typically suppresses expression, gene mutation and allelic loss. Under normal physiological conditions, DNMTs and TETs collaborate to maintain the balance of DNA methylation and demethylation. It has been demonstrated that aberrant DNA methylation is implicated in various diseases and plays an important role in atherosclerosis, including aberrant hypermethylation and hypomethylation [26]. Using whole-genome bisulfite sequencing, Zaina et al. [27] discovered that the atherosclerotic region of the aorta was hypermethylated across several genomic loci when compared to the corresponding healthy counterpart. However, there is genome-wide hypomethylation in atherosclerosis. Previous research has shown that hypomethylation of DNA predominates in atherosclerotic plaques [28]. In advanced human atherosclerotic lesions and ApoE knock-out mice lesions, genomic hypomethylation occurs with atherosclerosis [29]. These findings suggest that DNA methylation in atherosclerotic is a dynamic process, which is shown to be increased in early stages and decrease in late stages. The level of DNA methylation, on the other hand, is related not only to the stage of atherosclerosis, but also to the lesion grade of atherosclerosis. Using genome-wide DNA methylation sequencing, a positive correlation between DNA methylation and atherosclerotic lesion grade was discovered in atherosclerotic human aortas [30].

#### DNA hypermethylation and atherosclerosis

Increasing studies demonstrated that DNA methylation is regulated by inflammatory signaling pathways. For example, treatment of human umbilical vein endothelial cells (HUVECs) with pro-inflammatory stimuli, such as oxidized LDL (oxLDL), was shown to upregulate DNMT1 and lead to Kruppel-like factor 2 (KLF2) gene promoter methylation, resulting in KLF2 repression and endothelial inflammation accumulation [31]. Additionally, interleukin-6 (IL-6) can determine protein stabilization of DNMT1 and DNMT3B to induce changes in global and promoter-specific DNA methylation of genes [32]. Furthermore, inflammatory signaling pathways are regulated by DNA methylation. Inhibition of DNMT3b can increase the expression levels of forkhead box P3, transforming growth factor-β, and interleukin-10 and decrease the levels of interleukin-1β and interferon-gamma. In the process of regulating inflammatory factors, the generation mechanism of DNA methylation and the mechanism of DNA methylation in the three-dimensional structure of genome-related gene expression and regulation have not yet been elucidated. A recent survey of 542 human transcription factors found that 117 (22%) exhibited decreased binding to their motifs when methylated compared with unmethylated [33]. By preventing the binding of such transcription factors, DNA methylation can therefore impede transcription activation of CpG island promoters that contain their sequence recognition motifs.

In addition to inflammatory factors, DNA methylation is also affected by other stimuli. In vitro study revealed that ECs exposed to disturbed blood flow patterns have higher levels of DNMT1, resulting in DNA hypermethylation of their genome [34]. Obesity has been associated to an increased risk of serious cardiovascular or endocrinal diseases, such as atherosclerosis and stroke. In inguinal white adipose tissue of obese mice, Scara3 expression was reduced [35]. SCARA3 hypermethylation has been observed in patients with type 2 diabetes and atherosclerosis [35]. Moreover, findings from overweight/obese Korean subjects suggest an association between DNA hypermethylation at the TSPO-associated protein 1 antisense RNA 1 (TSPOAP1-AS1) promoter and overweight/obesity, as well as significant positive correlations between LDL cholesterol levels and TSPOAP1-AS1 DNA hypermethylation levels [36]. Whether hypermethylation in these promoter regions might be potential predictors of atherosclerosis is unclear; thus, this needs further investigation.

Atherosclerosis is a complex pathological process involving a variety of vascular wall cells and inflammatory cells. Endothelial dysfunction is the pathological basis of atherosclerosis, and it is accompanied by changes in vascular wall permeability. It leads to lipid accumulation, inflammatory cell infiltration, and smooth muscle cell migration and proliferation before developing into atherosclerosis. Gene expression changes caused by abnormal DNA methylation can lead to changes in cell phenotype and function. DNMT3b mediated hypermethylation of cellular repressor of E1A-stimulated genes (CREG) is observed in HUVECs treated with oxidized low-density lipoprotein (ox-LDL), a critical atherosclerogenic factor, result in CREG expression inhibition and endothelial dysfunction [37]. Homocysteine (Hcy), an independent risk factor for atherosclerosis, up-regulates phosphatase and tensin homologue on chromosome 10 (PTEN) methylation levels and enhances VSMCs proliferation, a primary pathological event in the development of atherosclerosis [38]. Hypermethylation of mitofusin-2 (MFN2), an important transmembrane GTPase in the mitochondrial outer membrane, can further facilitate VSMCs proliferation in Hcy-treated VSMCs. Increased c-Myc binding to DNMT1 promoter is a new and relevant molecular mechanism to contribute to MFN2 hypermethylation [39]. Hcy also plays a role in the inflammatory response and DNA methylation disorder in atherosclerosis, activating NF-κB-mediated vascular inflammatory response in human umbilical VSMCs via promoting SMAD7 promoter hypermethylation in a dose and time-dependent manner [40]. DNMT1 is a defining factor in macrophage inflammation both in vitro and in vivo. DNMT1 promotes macrophage M1 activation by suppressing the expression of Kruppel-like factor 4 (KLF4), and ApoE^−/−^ mice deficient Dnmt1 had ameliorated atheroma formation and suppressed plaque inflammation [41]. In a study of swine aortic endothelium isolated from disturbed flow regions, researchers discovered that hemodynamics increased DNA methylation of CpG islands within the KLF4 promoter via DNMT3a enrichment, with regional consequences for atherosclerosis [42]. DNMT3b accelerates atherosclerosis and may be associated with forkhead box P3 hypermethylation status in human peripheral blood regulatory T cells. In ApoE^−/−^ mice, Dnmt3b silencing attenuated atherosclerosis by decreasing lesion size and macrophage content while increasing collagen and smooth muscle cell content [43]. In both human atherosclerotic plaques and atherosclerosis patients, the SMAD7 promoter is hyper-methylated and it is positively related to homocysteine levels and carotid plaque scores [44]. In a sample of African-Americans, DNA methylation of AHRR, GFI1, and LRRC52 were associated with atherosclerosis after adjusting for cardiovascular diseases risk factors [45]. These findings suggest methylated SMAD7, AHRR, GFI1, and LRRC52 may be novel predicted biomarkers of atherosclerosis.

#### DNA hypomethylation and atherosclerosis

Atherosclerosis with DNA hypomethylation has been found in many studies in cell models, animals, and humans. Endothelial hypomethylation, which is induced by s-adenosylhomocysteine (SAH), the precursor of homocysteine [46, 47], led to augmented endothelial transmigration and decreased levels of aquaporin 1 and impaired water permeability, contributing to the development of atherosclerosis [48]. SAH levels by downregulating SAH hydrolase can also activate the expression of p66Shc, a key protein regulating oxidative stress, by reducing the expression of DNMT1 and the methylation of the p66Shc promoter. It then induces oxidative stress to damage endothelial function and contributes to the development of atherosclerosis in ApoE^−/−^ mice, suggesting that SAH-associated endothelial injury may contribute to the development of atherosclerosis [49, 50]. Inhibition of SAH hydrolase in ApoE^−/−^ mice epigenetically up-regulates Drp1 expression through repressing DNA methylation in endothelial cells, leading to vascular senescence and atherosclerosis [51]. Dynamin-related protein 1 (mdivi-1), a Drp1 specific inhibitor, alleviates atherosclerosis in ApoE^−/−^ mice by suppressing mito-ROS/NLRP3-mediated M1 polarization [52]. Ribonuclease 6 expression was upregulated in the peripheral blood and plaque tissues of atherosclerosis patients. In vivo and vitro study showed that hypomethylation of Ribonuclease 6 promoter aggravates atherosclerosis in mice, enhances proliferation and migration of oxLDL treated murine aortic VSMCs, and upregulated ROS content and inflammatory factor secretion levels in the cells [53]. Additionally, hypomethylation of the IL-6 promoter region was found in coronary heart disease subjects as compared with controls, and DNA hypomethylation in the IL-6 gene is associated with increased IL-6 gene expression in atherosclerosis patients [54, 55]. The expression of signaling lymphocytic activation molecule 7 (SLAM7) was significantly higher in advanced plaque than early atherosclerotic tissue as well as in the unstable plaques than in the stable plaques in atherosclerosis patients. High expression of SLAM7 promoted the secretion of proinflammatory cytokines and inhibited proliferation of VSMCs, which is a key regulator and could be a target of potential therapeutic intervention in atherosclerosis [56]. A genome-wide analysis found that the CpG site (cg11874627) at the promoter region of lipoprotein-associated phospholipase A2 is hypomethylated in vulnerable atherosclerotic lesions when compared with the non-vulnerable lesions with an increased expression upon inflammation [57]. Overall, these results suggested that aberrant DNA demethylation modifications (ie. p66Shc, Drp1, Ribonuclease 6, IL-6, SLAM7, and lipoprotein-associated phospholipase A2) contribute to atherosclerosis progression and its potential role in atherosclerosis as a therapeutic target.

TET1s deficiency exacerbates oscillatory shear flow-induced atherosclerosis [58]. DNA demethylating enzyme TET2 represses the upregulation of pro-inflammatory cytokines, chemokine, and inflammasome activation, thus preventing atherosclerosis [59, 60]. In line with both reports, hematopoietic or myeloid cell-specific TET2 deletion also aggravates cardiac dysfunction in heart failure, with activation of the NLRP3 /IL-1β pathway [61]. Recently, it was demonstrated that TET2 ameliorated atherosclerosis progression in ApoE^−/−^ mice via modulating Beclin1-dependent autophagic processes [62].

#### Histone modification in Atherosclerosis

Nucleosomes, which are made up of 147 base pairs of DNA and an octamer assembled by four core histones (H2A, H2B, H3, and H4), act as functional units of chromatin [12]. The N-terminal of histone extends beyond the nucleosome, and gene expression can be modified by acetylation, methylation, phosphorylation, ubiquitination, glycosylation, and ADP-ribosylation. This process is collectively defined as histone post‑translational modifications. Imbalances in histone modifications can lead to the development of cardiovascular diseases [63], and the loss of methylation and acetylation of histone H3 and H4 residues is a marker of atherosclerosis. Histone acetylation and methylation are the most studied modifications in inflammation and cardiovascular disease in these modifications [64].

#### Histone methylation

Histone methylation, which functions as maintenance and formation of heterochromatin structure, genomic imprinting, DNA repair, inactivation of X chromatin, and regulatory aspects of transcription, is a more stable epigenetic marker compared to histone acetylation. Histone methylation mainly occurs on lysine (k) or arginine (R) residues of histones H3 and H4. According to the methylation degree of each site, it can be divided into mono-methylation (me), di-methylation (me2), and tri-methylation (me3). Histone methylation is generally associated with transcriptional repression.

The histone methylation process is mainly catalyzed by two histone methyltransferases (HMT). Among them, histone lysine methyltransferase (HKMT) contains six histone lysine methyltransferase complexes (KMT1-6, listed in Table 1), whereas histone arginine methyltransferase (protein arginine methyltransferase, PRMT) contains PRMT1, 3, 5, 6, and CARM1. The histone demethylases are roughly divided into two families: LSD (Lysine-specific demethylase, contains LSD1 and LSD2) and JMJD (JmjC domain-containing family, contains KDM2, 3, 4, 5, 6). Generally, the methylation of different sites of histone H3 and H4 and the amount of methylation have great significance for the transcriptional regulation of genes. Among them, H3K9me3, H3K27me3, and H4K20me2/3 mediate transcriptional repression, whereas H3K4me1/2/3, H3K9me1, H3K27me1, H3K36me1/2/3, and H3k79me1/2/3 mediate transcriptional activation.

**Table 1:** Histone methyltransferase and demethyltransferase

		Class	Name	Site
Histone methyltransferase	histone lysine methyltransferase (HKMT)	KMT1	SUV39H1/2 G9a EHMT1 GLP SETDB1/2 CLLD8	H3K9
		KMT2	SET1A/B MLL1-5 ASH1L	H3K4 H4K20 H3K9
		KMT3	NSD1 SET2 SYMD2	H4K20 H3K36
		KMT4	DOT1L	H3K79
		KMT5	SET8 SUV4-20H1/2	H4K20
		KMT6	EZH1 EZH2	H3K27
		KMT7	SET7/9	H3K4
		KMT8	PRDM2 PRDM3 PRDM8 PPRDM16	H3K9
	protein arginine methyltransferase, PRMT		PRMT1	H4R3
			PRMT2	H3R8
			PRMT3	
			PRMT4( CARM1)	H3R2 H3R17 H3R26
			PRMT5	H3R8 H4R3
			PRMT6	H3R2 H4R3
			PRMT7	H4R3
			PRMT8	
			PRMT9	
Histone demethyltransferase	Lysine-specific demethylase, LSD	LSD1	KDM1A	H3K4 H3K9
		LSD2	KDM1B	H3K4
	JMJD (JmjC domain-containing family)	KDM2	JHDM1A (KDM2A) JHDM1B (KDM2B)	H3K36 H3K4
		KDM3	KDM3A KDM3B JMJD1C	H3K9
		KDM4	KDM4A KDM4B KDM4C KDM4D	H3K9 H3K36
		KDM5	KDM5A KDM5B KDM5C KDM5D	H3K4
		KDM6	UTX JMJD3 UTY	H3K27
		KDM7	JHDM1D PHF8	H3K9 H4K20

The development of atherosclerosis was significantly associated with inflammatory factors, which were secreted from the M1 pro-inflammatory phenotype. Transient receptor potential A1, a calcium-permeable non-selective cation channel, is overexpressed in atherosclerosis. It can change the H3K27 trimethylation level in macrophages and regulate the macrophages toward an inflammatory phenotype [65]. In monocytes, reduced methylation of H3K9 and H3K27 was shown in inflammatory cells [66]. The plasma concentrations of monocyte chemoattractant protein 1 (MCP1) in CD14^+^ monocytes from coronary heart disease patients were significantly upregulated, and the H3K9 tri-methylation of the MCP1 promoter was decreased [67]. MCP1 affects the chemotaxis of monocytes and is a key chemokine closely related to the development of atherosclerosis.

Upregulation of histone lysine methyltransferase SETDB2, a member of the KMT1 family, was observed in proinflammatory M1, whereas deficiency of SETDB2 in hematopoietic cells promoted vascular inflammation and accelerated atherosclerosis [68]. Histone H3K27 methyltransferase Ezh2 represses Socs3, the suppressor of cytokine signaling, to increase macrophage inflammatory responses. Myeloid-specific Ezh2 deficiency can reduce macrophage foam cell inflammatory response with reduced production of nitric oxide, IL-6, and IL-12, contributing to reduced atherosclerosis in mice [69]. In VSMC-specific disruptor of telomeric silencing 1-like (Dot1l) conditional knock-out mouse model, Dot1l and its uniquely induced H3K79me2 directly regulate the transcription of Nf-κB, result in increased expression of CCL5 and CXCL10 [70]. Dot1l deficiency in mice lowers atherosclerotic plaque stability and promotes inflammatory plaque macrophages activation by controlling lipid biosynthesis gene programs [71]. These findings suggest DOT1L as a potential therapeutic target for atherosclerosis.

On the other hand, lysine demethylase KDM4A/JMJD2A directly targets oxLDL-induced M1 polarization of macrophages independent of NF-κB and HIF activation, two signals critical for pro-inflammatory activation of macrophages [72]. LPS treatment promoted JMJD3 expression and enhanced JMJD3 nuclear accumulation in HUVECs. JMJD3 attenuated the methylation status in the promoter region of target genes, culminating in target gene expression [73].

#### Histone acetylation and deacetylation

The changes in histone acetyl groups have been widely considered an epigenetic marker of atherosclerosis. Acetylation results from the transfer of an acetyl group from acetyl-CoA to the ε-amino side chain of lysine by lysine acetyltransferases (KAT). This process can be reversed by KDAC. Adding acetyl groups to histone structure reduces its positive charge and affinity for negatively charged DNA, thus increasing the transcriptional accessibility of chromatin [74, 75]. Histone acetylation is the most widely studied form of histone modification. Histone acetyltransferases (HAT) and histone deacetylases (HDACs) were identified in the mid-1990s to late 1990s. HATs and HDACs were renamed KATs and lysine deacetylases (KDACs), respectively, to differentiate from non-histone acetylation. These two enzymes regulate histone acetylation level and gene transcription by reversible modification of histone. KATs are classified into three classes: GCN5 (GCN5-related N-acetyltransferases family; contains GCN5 and PCAF), MYST (MOZ, MORF, Ybf2/Sas3, Sas2, and Tip60), and P300 (CBP and P300). KDACs are classified into four classes: class 1 (histone deacetylase (HDAC) 1, 2, 3, 8), class 2 (HDAC 4, 5, 6, 7, 9, 10), class3 (SIRT 1–7), and class4 (HDAC11) [76]. Class1, 2, and 4 KDACs are classical Zn-dependent deacetylases, whereas the class 4 KDAC is NAD-dependent sirtuin deacetylases. In human macrophages, LPS induces the recruitment of p300 and enhances histone acetylation at the sites of active transcription within proximal promoter of NADPH oxidase 5 gene, suggesting that pharmacological targeting of epigenetic-based pathways that control NADPH oxidase 5 expression might be a noteworthy novel therapeutic strategy in atherosclerosis [74].

##### Class I KDACs

The increasing number of evidence has shown that HDACs are involved in the phenotype switch of VSMCs proliferation and migration. Recently study found that HDAC1, an important modulator, was critical for the migration and phenotypic switch of aortic VSMCs [77]. Regulatory factor X1 deficiency in CD14 + monocytes facilitated H3 and H4 acetylation and H3K9 tri-methylation in the MCP1 promoter region and contributed to MCP1 overexpression via reducing the recruitments of HDAC1 and suppressor of variegation 3–9 homolog 1 (SUV39H1) [78]. In endothelial cells, histone deacetylase 2 (HDAC2) protects against endothelial dysfunction and atherogenesis [79]. Endothelial-mesenchymal transition (EndMT) is a vital factor of plaque instability in atherosclerosis. HDACs are closely related to vascular endothelium stability and EndMT in atherosclerosis. HDAC3, an essential pro-survival molecule, is essential for differentiating endothelial progenitors. When HDAC3 is knocked down, ApoE^−/−^ mice develop atherosclerosis, and their vessel ruptures [80]. HDAC3 also affects EndMT in atherosclerosis. In ApoE^−/−^ mice and HUVECs, HDAC3 inhibitor suppresses EndMT via modulating inflammatory [81]. HDAC3 protects against atherosclerosis through inhibition of inflammation via the microRNA-19b/PPARγ/NF-κB axis in ox-LDL treated HUVECs and ApoE^−/−^ mice [82].

##### Class II KDACs

HDAC4 is a key regulator and participates in proliferation and migration in various cell types [83–85]. HDAC4 can promote VSMC proliferation and migration and can be inhibited by interfering with HDAC4 [86]. Besides, HDAC4 was also involved in vascular calcification (VC). Recently, Abend et al. [87, 88] found that HDAC4 was upregulated early in VC and involved in vascular calcification and inflammatory response in VSMCs. VC is now widely known to be an active process occurring in VSMCs and is characterized by calcium deposition inside arteries. It is also associated with the morbidity and mortality of atherosclerosis [87, 89]. Whereas HDAC5 acts as a pro-inflammatory molecule in VSMCs, which is mediated by Nox4-dependent ROS production, and phosphatidylinositol 3-kinase (PI3K)/AKT pathways [90]. HDAC6, on the other hand, is upregulated by atherogenic stimuli via posttranslational modifications and is a critical regulator of CSEγ expression in vascular endothelium. In endothelial cells treated with oxLDL, the expression of CSEγ and H2S production are decreased, and it leads to endothelial dysfunction. The vascular endothelium is protected by cystathionine γ-lyase via inhibition of HDAC6 activity [91]. Targeting HDAC6 attenuates nicotine-induced macrophage pyroptosis via NF-κB/NLRP3 pathway in atherosclerosis [92].

Atherosclerosis-prone mice showed reduced EndMT and significantly reduced plaque area while endothelial-specific HDAC9 knockout while endothelial-specific HDAC9 controlled EndMT and the atherosclerotic plaque phenotype [93]. Malhotra et al. found that HDAC9 is associated with abdominal aortic calcification and affects VSMCs phenotype. In human aortic VSMCs, overexpression of HDAC9 promoted calcification and reduced contractility, whereas decreased expression of HDAC9 inhibited calcification and enhanced cell contractility [94]. HDAC9 promotes endothelial-mesenchymal transition and an unfavorable atherosclerotic plaque phenotype [93]. VSMCs calcification could be inhibited by HDAC inhibitors, such as apicidin, trichostatin, vorinostat, and tubacin [95–97].

##### Class III KDACs

SIRT6 (Sirtuin 6) is a nuclear deacetylase and plays a key role in regulating VSMCs senescence and atherosclerosis. In human and mouse plaque VSMCs, SIRT6 protein expression is reduced and regulated by CHIP. SIRT6 regulates telomere maintenance and VSMCs lifespan and then inhibits atherogenesis, which is dependent on its deacetylase activity. Endogenous SIRT6 deacetylase is an important inhibitor of VSMCs senescence and atherosclerosis [98].

### Targeting DNA methylation and histone modification in the treatment of atherosclerosis

Extensive epigenetic modifications contribute to the development and progress of atherosclerotic plaque. Clinically, aspirin absorption leads to ABCB1 lower methylation in intracranial artery stenosis patients [99]. Folic acid (the deficiency of which increases homocysteine levels) is an atherosclerotic drug that induces endothelial dysfunction, accelerates atherosclerotic pathological processes, and increases DNA methylation of vascular peroxidase 1, MCP1, and vascular endothelial growth factor in high-fat diet-fed ApoE knockout mice [100, 101]. Therefore, epigenetic modification, including DNA methylation and histone post-translational modification, is considered to be a promising method for the treatment of many diseases, including atherosclerosis.

#### Treatment strategies targeting DNA methylation

DNMT inhibitors are among the first epigenetic drugs to be used in cancer therapeutics [102]. Drugs targeting DNMT include cytosine analogs, oligonucleotide drugs, DNA binder, and S-adenosylmethionine competitors. Cytosine analogs can be irreversibly incorporated into DNA during DNA synthesis. When DNMT tries to catalyze DNA methylation, these cytosine analogs DNMT can covalently bind to DNMT so that DNMT cannot be detached from chromatin, resulting in inhibition of activity. Presently, there are two FDA-approved cytosine analogs DNMT, namely 5-Azacytidine (5-Aza-C) and 5-Aza-CdR (trade name, decitabine). 5-Aza-C is used to treat myelodysplastic syndromes. It interferes not only with DNA methylation but also mRNA synthesis, thus having a relatively strong toxicity. Pharmacological upregulation of PTEN by 5-Aza-C reduces plaque area and preserves SMC contractile protein expression in vivo [103]. 5-Aza-CdR (trade name, decitabine), which is indicated for myelodysplastic syndromes and acute myeloid leukemia, only interferes with deoxyribonucleic acid without interfering with the nucleic acid. Inhibiting DNA methylation by 5-Aza-2'-deoxycytidine ameliorates atherosclerosis by suppressing macrophage inflammation [104]. Interestingly, increasing studies identified that 5-Aza-CdR effectively inhibits atherosclerosis development in several well-established animal models of atherosclerosis, including diet-induced atherosclerosis in ApoE^−/−^ mice, LDLr^−/−^ mice, and ApoE^−/−^ mice undergoing carotid partial ligation [104–106].

However, pharmacological editing of global DNA methylation lacks specificity and may result in adverse reactions, such as autoimmune disorders [107]. In human atherosclerotic arteries, there was a negative correlation between DNMT3B and CREG expression levels, indicating blocking CREG methylation may represent a novel therapeutic approach to treat ox-LDL-induced atherosclerosis [37]. Recently, Ziltivekimab, a fully human monoclonal antibody directed against the IL-6 ligand, was shown to markedly reduced biomarkers of inflammation and thrombosis relevant to atherosclerosis in RESCUE trial [108, 109]. Furthermore, ziltivekimab-mediated IL-6 ligand inhibition is associated with a lower neutrophil–lymphocyte ratio, which independently predicts atherosclerotic events and is a potential biomarker for residual inflammatory risk, suggesting that it may disrupt multiple atherogenic inflammatory pathways [110]. On the other hand, Drp1 inhibition reduced macrophage burden, oxidative stress, and advanced calcified atherosclerotic plaque in aortic roots of diabetic ApoE^−/−^ mice, as well as inflammatory cytokine production in human macrophages. Mdivi-1, a Drp1 specific inhibitor, was identified as a novel small molecule proprotein convertase subtilisin/kexin type 9 (PCSK9) inhibitor, which represents a cornerstone of cardiovascular prevention [111, 112].

Oligonucleotide drugs**,** such as MG98**,** target the active catalytic pocket of DNMT and prevent DNMT from binding to the promoter of a specific gene to inhibit its DNA methylation. DNA binders, such as SGI-1027, target cofactor-binding sites of DNMTs. Although the relationship between oligonucleotide drugs, DNA binder, and disease has not been studied, they may become an alternative approach, as they have low cytotoxicity without being incorporated into DNA. Additionally, several Chinese herbal medicines show a potential regulatory effect on DNA methylation in atherosclerosis [1]. Some herbs and herbal compounds, such as curcumin, geniposide, and resveratrol, have also shown promise in modulating epigenetic enzymes in vascular cells and atherosclerosis [26, 38, 113, 114]. Although the current literature has proven that some of the naturally occurring non-nucleoside DNMTi is capable of inhibiting atherosclerosis in mice, it is unknown how far their specific mechanism of action and the DNMTs inhibiting effects of these compounds contribute to their atheroprotective effects. Only a few drugs currently used to treat neuropsychiatric disorders have a direct effect on histone-modifying enzymes or on DNMTs. Importantly, global DNA methylation targeting cytidine analogues and non-intercalating methyltransferase inhibitors are FDA approved for the treatment of certain cancers [115]. DNA methylation is dynamic and reversible, with individual differences and specificity in time and space. Therefore, the design of specific drugs for DNA methylation markers of specific individuals and specific stages of the disease has become a challenge for future drug development.

Thus, targeting DNA methylation pathways may represent a promising avenue for therapy in atherosclerosis, similar to current clinical uses of DNA-hypomethylating agents in leukemia. However, further work is needed to decipher cell- and gene-specific DNA methylation changes (especially in humans) and to determine if treatment with DNMT inhibitors has disparate effects at distinct stages of the disease.

#### Treatment strategies targeting histone modification

Histone methylation is usually associated with transcriptional inhibition. Histone methylation inhibitors (HTMi), when compared to other epigenetic inhibitors, have not been extensively studied and remain an untapped resource. GSK126 is a potent histone methylation inhibitor, which is highly selective for the histone N-methyltransferase EZH2. Additionally, it can inhibit H3K27me3 and severely attenuate the expression of proinflammatory genes at both mRNA and protein levels [116, 117].

Regarding potential modulators of histone acetylation, garcinol and anacardic acid are natural compounds that show histone acetyltransferase inhibitors (HATi) activity. Garcinol, a polyisoprenylated benzophenone derived from the *Garcinia indica* fruit, was used to investigate the role of histone acetylation in the regulation of early growth response protein 1 (EGR1) gene [118, 119]. Recently, a new anacardic acid analogue, MG149, has been developed as an effective and selective inhibitor of the histone acetyltransferases (HATs) MYST family (Tip60, KAT5, and MOZ). MG149 can inhibit the NF-κB pathway, which is involved in the expression of various pro-inflammatory cytokines and plays a key role in inflammatory diseases, such as atherosclerosis [120, 121].

Histone acetylation is associated with open chromatin and gene transcription, which can be influenced by HDACs through counteracting HATs. Nowadays, HDAC inhibitors (HDACi), potential modifiers of histone acetylation, have been approved for the treatment of hematological malignancies, even though their application in atherosclerosis has not been investigated in clinical trials [122]. The pharmacological effects of HDACi are mediated through the reactivation of silenced genes by preventing histone deacetylation on target gene promoters. HDACi is mainly divided into four groups, including cyclic peptides, aliphatic acids, and benzamides [123]. Vorinostat (an HDAC inhibitor), also known as suberoylanilide hydroxamic acid (SAHA), is a compound derived from the hydroxamic acid class. It has been approved for T-cell cutaneous lymphoma therapy by the FDA, and it affects all classes of HDACs except class III. SAHA has been reported to decrease atherosclerotic lesion size in ApoE deficient mice in a KLF2-dependent manner [75, 124]. Trichostatin A (TSA), another specific HDACi, exacerbates atherosclerosis via increasing acetylation at the scavenger receptor CD36 promoter region, tumor necrosis factor (TNF)-alpha, and vascular cell adhesion molecule-1 (VCAM-1) and decreasing IL-6 and IL-1beta expressions in Ldlr^−/−^ mice [125, 126]. Recently, TSA was found to target C/EBPα/PPARγ axis and induce acetylation of C/EBPα to alleviate atherosclerosis [127]. These varieties effect of TSA in atherosclerosis may attribute to its nonspecific, as TSA have an inhibitory effect toward HDAC I, IIA, and IIB. This phenomenon suggests that HDAC inhibition-dependent and -independent mechanisms must be explored when assessing the pharmacological effects of HDACi. HDACi are a promising class of anti-inflammatory drugs. Recently, an efficient drug delivery system carrying the class I/IIa selective HDACi, such as valproic acid (VPA), was developed to circumvent common disadvantages of free drug administration, e.g., short half-life and side effects. Additionally, it exhibits anti-inflammatory effects in primary human macrophages and is able to attenuate the lipopolysaccharide-induced inflammatory response [128]. Treatment with TMP195, a selective inhibitor of Class IIa HDAC, reduced critical inflammatory pathways and mitigated atherogenesis in advanced stage atherosclerosis, thereby offering a novel therapeutic strategy for reducing the consequence of vascular inflammation [129]. It is well known that the majority of existing or clinically available HDAC inhibitors are generic [130]. The development of selective HDACi could reduce the side effects of other target activities, such as the potential generic toxicity of HDAC6 [131]. Romidepsin (FK228) is a selective inhibitor against HDAC1/2 with anti-inflammatory properties and an effect on SMCs proliferation through regulating the deacetylation of several transcription factors (krüppel-like factor 5, CREB binding protein) [132, 133]. In ApoE^−/−^ mice, HDAC3 specific inhibitor RGFP966 alleviated atherosclerotic lesions and inhibited EndMT of the atherosclerotic plaque [81]. Bossche et al. [134] found that inhibition of HDAC3 had the atherogenic protective effect of pan-HDAC inhibitors using specific HDAC inhibitor. Due to a partial reduction in M1 activation without an increase in foam cells, HDAC inhibition in macrophages, particularly HDAC3, displayed anti-atherogenic effects. In high-fat diet-fed ApoE^−/−^ mice, Romidepsin-enhanced STAT3 acetylation epigenetically modulates VCAM-1 expression to suppress atherosclerosis [135]. As DNA methylation is accompanied by histone deacetylation, the combination of DNA methylation inhibitors and histone deacetylation inhibitors in the treatment of inflammatory diseases have become popular. Considering that existing chemotherapies, and additional drugs in development that modulate epigenetic silencing may increase risk of myocardial infarction, therapies that target specific cells may be an alternative to atherosclerosis treatment. From human genetics, ZEB2, a master regulator of EndMT, was found to be a coronary artery disease associated gene, and ZEB2 regulates SMC phenotypic transition through epigenetic inhibition of TGFβ and NOTCH signaling in atherosclerosis [136].

#### Nanomaterials for direct epigenetic therapy for atherosclerosis

Targeting DNA methylation or histone modification may represent a promising avenue for atherosclerosis therapy. However, as stated in the article, the main challenge of epigenetic therapy is the possibility of side-effects due to the fact that many targets of epigenetic-drugs are ubiquitously expressed, and that some epigenetic -drugs had a poor bioavailability, low stability and a short half-life [137]. Nanomaterials, which include organic nanoparticles (e.g. polymeric nanoparticle, liposomes, micelles, and high-density lipoprotein nanoparticle), and inorganic nanoparticles (e.g. gold nanoparticles, Fe_3_O_4_, mesoporous silica nanoparticles, and CuS) have been shown to be effective for therapy and diagnosis for atherosclerosis [138, 139]. Indeed, in recent years, a large number of nanoparticles have been developed with physical and chemical characteristics that allow them effectively deliver the epigenetic-drugs to the diseased cells and control their release [reviewed in [137]]. Nanoparticles comprising gelatinase with polyethylene glycol (PEG) and poly-ε-caprolactone) to specifically deliver DAC, for example, greatly inhibited tumor growth in mouse gastric cancer xenograft model [140]. Polymeric nanoparticles functionalized with histone deacetylase inhibitors (iHDACs) resulted in optimal iHDACs release in mesothelioma cancer, leading in a significant reduction in the weight of the tumor without toxicity [141]. Liposomes, on the other hand, have been used as nano-carriers of epigenetic-drugs: PEG-functionalized liposomes transport and release some anti-tumor drugs more efficiently, including the HDAC inhibitors SAHA, PXD101 and TSA [142].

## Conclusion

The chronic progressive nature of atherosclerosis has highlighted atherosclerosis heterogeneity and the fact that specific cell types in the complex milieu of the plaque are, by far, not the only initiators and drivers of atherosclerosis. Instead, the ubiquitous effects of cell type are tightly controlled and directed by the epigenetic signature, which, in turn, is affected by many proatherogenic stimuli, including LDL, proinflammatory, and physical forces of blood circulation. Therefore, defining the role of epigenetics in vascular pathogenesis using human atherosclerotic tissue or animal atherosclerotic models is complicated by the dynamic nature of the disease and tissue heterogeneity. The ubiquitous effects of epigenetic changes on varieties of cell types limit clinical application in disease specificity or treatment. Therefore, systematic detection of epigenetic changes over different time periods allows us to develop comprehensive therapeutic strategies. Another problem faced is that when multiple post-translational modifications target the same amino acid residue (for example, lysine residues), there may be competitive antagonism between different modifications. How can atherosclerosis-related cells seek common ground while reserving difference? Further research is required to appropriately place the epigenetic modifiers in the treatment algorithm of atherosclerosis.

## Data Availability

Not applicable.

## Abbreviations

CpG: 5’-Cphosphate-G-3’
CG: Cytosine guanine
DNMTs: DNA methyltransferases
ECs: Endothelial cells
EndMT: Endothelial-mesenchymal transition
HAT: Histone acetyltransferases
Hcy: Homocysteine
HDACs: Histone deacetylases
HKMT: Histone lysine methyltransferase
HMT: Histone methyltransferases
HUVECs: Human umbilical vein endothelial cells
IL-6: Interleukin-6
KLF4: Kruppel-like factor 4
LDL: Low-density lipoprotein
MCP1: Monocyte chemoattractant protein 1
oxLDL: Oxidized LDL
SAH: S-adenosylhomocysteine
SIRT6: Sirtuin 6:
TET: Ten-eleven translocation protein
VC: Vascular calcification
VSMCs: Vascular smooth muscle cells
5-Aza-C: 5-Azacytidine
5-hmC: 5-Hydroxymethylcytosine
5mC: 5-Methylcytosine
